# Identification of mitophagy‐related biomarkers in osteoarthritis

**DOI:** 10.1002/ame2.12416

**Published:** 2024-05-08

**Authors:** Shiqiang Ruan, Dongxu Tang, Yanfei Luo, Hao Song

**Affiliations:** ^1^ Department of Orthopaedics Surgery the First People's Hospital of Zunyi City (The Third Affiliated Hospital of Zunyi Medical University) Zunyi China

**Keywords:** diagnosis, immune cell infiltration, machine learning, mitophagy, osteoarthritis (OA), quantitative real‐time polymerase chain reaction (qPCR)

## Abstract

**Background:**

Osteoarthritis (OA) is a common joint disease, and existing drugs cannot cure OA, so there is an urgent need to identify new targets. Mitophagy plays an important role in OA; however, the role of mitophagy in the OA immune system is not yet clear.

**Methods:**

In this study, differential analysis and enrichment analysis were used to identify mitophagy‐related genes (MRGs) with differential expression in OA and the functional pathways involved in OA. Subsequently, two machine learning methods, RF and LASSO, were used to screen MRGs with diagnostic value and construct nomograms. At the same time, the relationship between mitophagy and OA immune response was explored by immunoinfiltration analysis.

**Results:**

Forty‐three differentially MRGs were identified in OA, of which six MRGs (GABARAPL2, PARL, GABARAPL1, JUN, RRAS, and SNX7) were associated with the diagnosis of OA. The ROC analysis results show that these 6 MRGs have high predictive accuracy in the diagnosis of OA. In immune infiltration analysis, we found that the abundance of significantly different immune cells in OA was mostly upregulated. In addition, the expression of diagnostic‐related MRGs is correlated with changes in the abundance of immune cells in OA.

**Conclusion:**

This study demonstrates that six MRGs can be used as diagnostic biomarkers. The expression of diagnostic‐related MRGs is correlated with changes in the abundance of immune cells in OA. At the same time, mitophagy may affect the immune microenvironment of OA by regulating immune cells, ultimately leading to the progression of OA.

## Graphical abstract

Differential analysis of OA‐related datasets identified 43 mitophagy‐related genes (MRGs) differentially expressed in OA. Subsequently, six MRGS (GABARAPL2, PARL, GABARAPL1, JUN, RRAS and SNX7) associated with OA diagnosis were identified using RF and LASSO regression analysis. ROC analysis showed that the six MRGS had high predictive accuracy for the diagnosis of OA. Given the high predictive accuracy of the six diagnosis‐related MRGS, the nomogram model we constructed performed similarly to the ideal model in clinical practice. In the immune infiltration analysis, we found that the significantly different immune cell abundances were mostly upregulated in OA. In addition, there is a correlation between the expression of diagnosis‐related MRGs and the changes in the abundance of immune cells in OA, suggesting that mitophagia may affect the immune microenvironment of OA by regulating immune cells, ultimately leading to the progression of OA. In addition, the results of qPCR experiments showed significant differences in the expression of six MRGs between OA and normal tissues, consistent with the results of bioinformatics analysis. The results of this study may provide new insights into the role of mitophagy in OA and provide new targets for the diagnosis and treatment of OA.

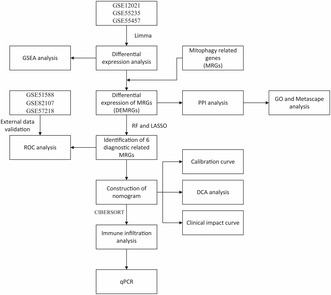

## 1 INTRODUCTION

Osteoarthritis (OA) is a common degenerative joint disease characterized by cartilage degeneration and synovial inflammation.^1^, ^2^ The occurrence of OA is related to various risk factors, including aging, obesity, joint damage genetics, etc.^3^ OA has no obvious symptoms in the early stage and requires radiological evidence to be diagnosed.^4^ When patients experience pain symptoms in the later stage of OA, irreversible damage may have occurred to their joints.^5^ At present, joint replacement surgery is an effective treatment for OA, and drugs that can cure OA have not been developed.^6^ Therefore, there is an urgent need to identify new biomarkers to improve the early diagnosis and treatment of OA.

Numerous studies have confirmed that mitophagy plays an important role in OA, and regulating mitophagy levels may be a promising strategy for OA.^7^ Mitophagy can significantly affect bone metabolism disorders and the proliferation, differentiation, and functional maintenance of osteoblasts.^8^ Dysfunction of osteoblasts will cause abnormal bone remodeling and changes in bone microstructure, leading to the development of OA.^9^ Mitophagy plays a dual role in OA. Shin et al. found that Pink1 can induce cartilage degeneration in OA by mediating mitophagy.^10^ Hu et al.'s study showed that HIF‐1 α can alleviate the symptoms of OA by enhancing mitophagy.^11^ Given the recently established importance of mitophagy in OA, it has great potential as a therapeutic target for improving OA.^12^, ^13^

The immune response plays an important role in the progression of OA.^14^ According to reports, T cells and activated macrophages are closely related to the progression of OA disease and serve as pain mediators in OA.^15^ Early immune response is a driving factor in the pathogenesis of OA, which can gradually cause degenerative changes in joints and alter their microenvironment.^16^ Mitophagy plays an important role in the homeostasis of the innate immune system.^17^ However, the role of mitophagy in the OA immune system is not yet clear.

In this study, we first conducted differential analysis and enrichment analysis to identify MRGs with differential expression in OA and the functional pathways involved in OA. Subsequently, we used two machine learning methods, RF and LASSO, to screen MRGs with diagnostic value. We constructed a nomogram based on diagnostic‐related MRGs to evaluate their ability in clinical application of OA. In addition, we explored the relationship between mitophagy and OA immune response through immune infiltration analysis. Finally, the expression of diagnostic‐related MRGs in OA and normal tissues was verified through qPCR experiments. The results of this study may provide new insights into the role of mitophagy in OA and provide new targets for the diagnosis and treatment of OA.

## 2 METHODS

### 2.1 Data acquisition

Six gene expression array data including control and OA patients were obtained from the public database GEO (http://www.ncbi.nlm.nih.gov/geo), including GSE12021, GSE55235, GSE55457, GSE51588, GSE82107, and GSE57218. The ‘SVA’ package was used to remove batch effects from the GSE12021, GSE55235, and GSE55457 datasets, and merged into one dataset (29 controls and 30 OA patients) as the training dataset for this study (Table 1).

**TABLE 1:** The information of the datasets obtained from GEO.

Dataset	Platform	OA	Control
GSE12021	GPL96	10	9
GSE55235	GPL96	10	10
GSE55457	GPL96	10	10
GSE51588	GPL13497	40	10
GSE82107	GPL570	10	7
GSE57218	GPL6947	33	7

### 2.2 Identification of differentially expressed genes in OA

Firstly, differential analysis was performed on the training dataset using the R package ‘limma’ to identify differentially expressed genes (DEGs) in the control and OA. Genes with an adjusted *p* value less than 0.05 were identified as DEGs in OA. R packages ‘pheatmap’ and ‘ggplot2’ were used to draw heatmaps and volcano plots.

### 2.3 Functional enrichment analysis

The R package ‘clusterProfiler’ was used to perform GSE analysis, with ‘c2. cp. kegg. v7.4. symbols. gmt’ as the reference gene set. Pathways were visualized with adjusted *p* values less than 0.05 using the R package ‘enrichplot’.

Using the R package ‘clusterProfiler’ to perform GO analysis, pathways with a *p* value less than 0.05 are considered statistically significant. The GO analysis results are presented using the R package ‘ggplot2’. Metascape analysis was based on an online platform (https://metascape.org/gp/index.html) to obtain pathways and biological functions enriched in the target gene sets.

### 2.4 Identification of differentially expressed mitophagy‐related genes in OA

One hundred and thirty‐seven MRGs were obtained from previous research data,^18^ and the gene list is included in Supplementary Material 1. The intersection between DEGs and MRGs was used to obtain differentially expressed MRGs in OA (DEMRGs). The R package ‘ggpubr’ was used to draw a box diagram. In addition, we obtained the protein–protein interaction (PPI) relationships of DEMRGs based on the STRING database (https://cn.string‐db.org/). We used the Analyze Network plugin in Cytoscape to calculate the Degree of DEMRGs in the PPI network and visualize it.

### 2.5 Identification of diagnostic‐related MRGs in OA

This study used Random forest (RF) and LASSO regression algorithms to identify diagnostic‐related MRGs in OA. Firstly, the RF algorithm (R packet ‘randomForest’) was used to evaluate the importance of MRGs. Genes with an importance score greater than 1.5 were used as candidate diagnostic biomarkers for OA. Then, LASSO regression analysis (R package ‘glmnet’) was used to analyze candidate diagnostic‐related genes to identify diagnostic‐related MRGs in OA. The R package ‘pROC’ was used to perform ROC analysis to evaluate the predictive accuracy of diagnostic genes. GSE51588, GSE82107, and GSE57218 were used as validation datasets for ROC analysis of diagnostic genes, respectively.

In addition, to further evaluate the value of diagnostic‐related MRGs in clinical guidance of OA, we constructed a nomogram model using the ‘rms’ package based on diagnostic‐related MRGs. Calibration curves, DCA analysis, and clinical impact curves are used to verify the diagnostic accuracy of the nomogram model. The ‘SVA’ package was used to remove batch effects from the training dataset, GSE51588, GSE82107, and GSE57218 datasets, and merge them into one dataset (53 controls and 113 OA patients) as input data for constructing the nomogram model.

### 2.6 Immune infiltration analysis

The R package ‘e1071’ was used to perform CIBERSORT analysis to elucidate the immune landscape of 22 immune cells in control samples and OA patients. The R package ‘pheatmap’ was used to plot the abundance heatmap of immune cells (with an abundance greater than 0 in more than half of the samples). We calculated the differences in immune cells between OA and control samples using wilcox.test. We used Pearson's method to calculate the correlation between diagnostic‐related MRGs and 22 types of immune cells, in order to explore the impact of MRGs in the OA immune microenvironment.

### 2.7 QRT‐PCR analysis

Total RNA was isolated using Trizol reagent (Life Technologies). Single‐stranded cDNA was prepared from 1 μg of total RNA using reverse transcriptase with oligo‐dT primer according the manufacturer's instructions (Promega, USA). The primers used are listed in Table S1. Values were normalized to GAPDH mRNA levels and calculated by 2^−ΔΔCt^ qPCR analysis, providing primer sequences (Supplementary Material 2).

### 2.8 Statistic analysis

In this study, the wilcox.test was used to calculate the differences between the two groups. A *p* value less than 0.05 was considered statistically significant (*p < 0.05, **p < 0.01, ***p < 0.001).

## 3 RESULTS

### 3.1 Screen DEGs in OA

**FIGURE 1:**
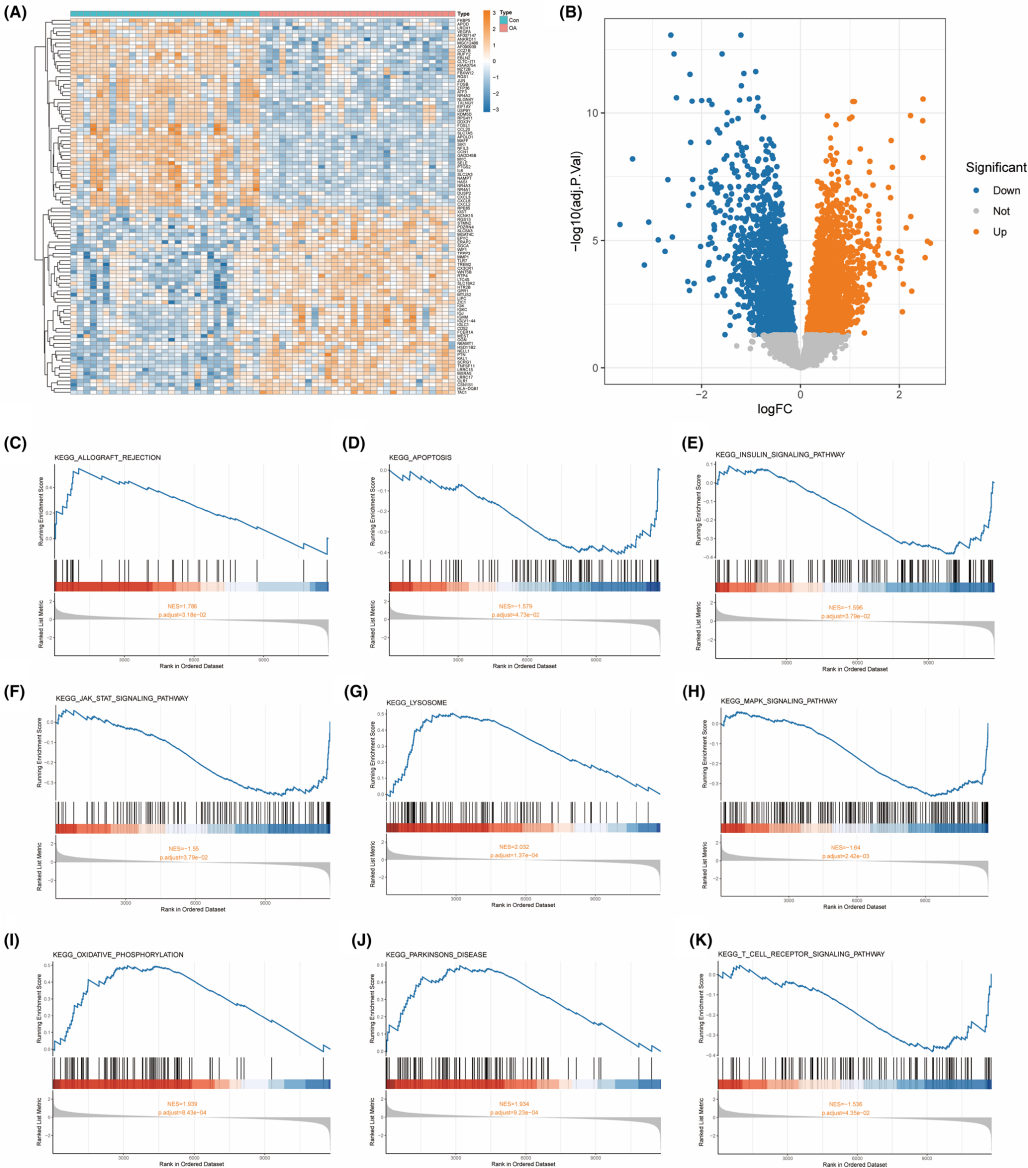
Expression of DEGs in OA and GSEA analysis results. (A), The expression heatmap of DEGs in the merged dataset. Yellow represents genes upregulated in OA, while blue represents genes downregulated in OA. Red represents OA, while blue represents normal. (B), Volcano plot of DEGs in the training dataset. (C–K), Nine KEGG pathways significantly enriched in OA.

OA‐related datasets (GSE12021, GSE55235, and GSE55457) were merged as training datasets for subsequent analysis. The results of differential analysis showed that a total of 4420 genes were differentially expressed in OA and normal tissues, including 2227 upregulated genes and 2193 downregulated genes (Supplementary Material 3). In Figure 1A, the expression landscape of the top 50 upregulated genes and the top 50 downregulated genes in OA and normal tissues is displayed. The volcano plot showed the expression status of all genes in the merged dataset (Figure 1B).

GSEA analysis shows (Figure 1C–K) that the DEGs in the OA merged dataset mainly participate in the Allograft rejection, Apoptosis, Insulin signaling pathway, JAK STAT signaling pathway, Lysosome, MAPK signaling pathway, Oxidative phonology, Parkinsons disease, and T cell receiver signaling pathway. The remaining 10 significantly enriched pathways are shown in Supplementary Material 4.

### 3.2 Identification of DEMRGs in OA

**FIGURE 2:**
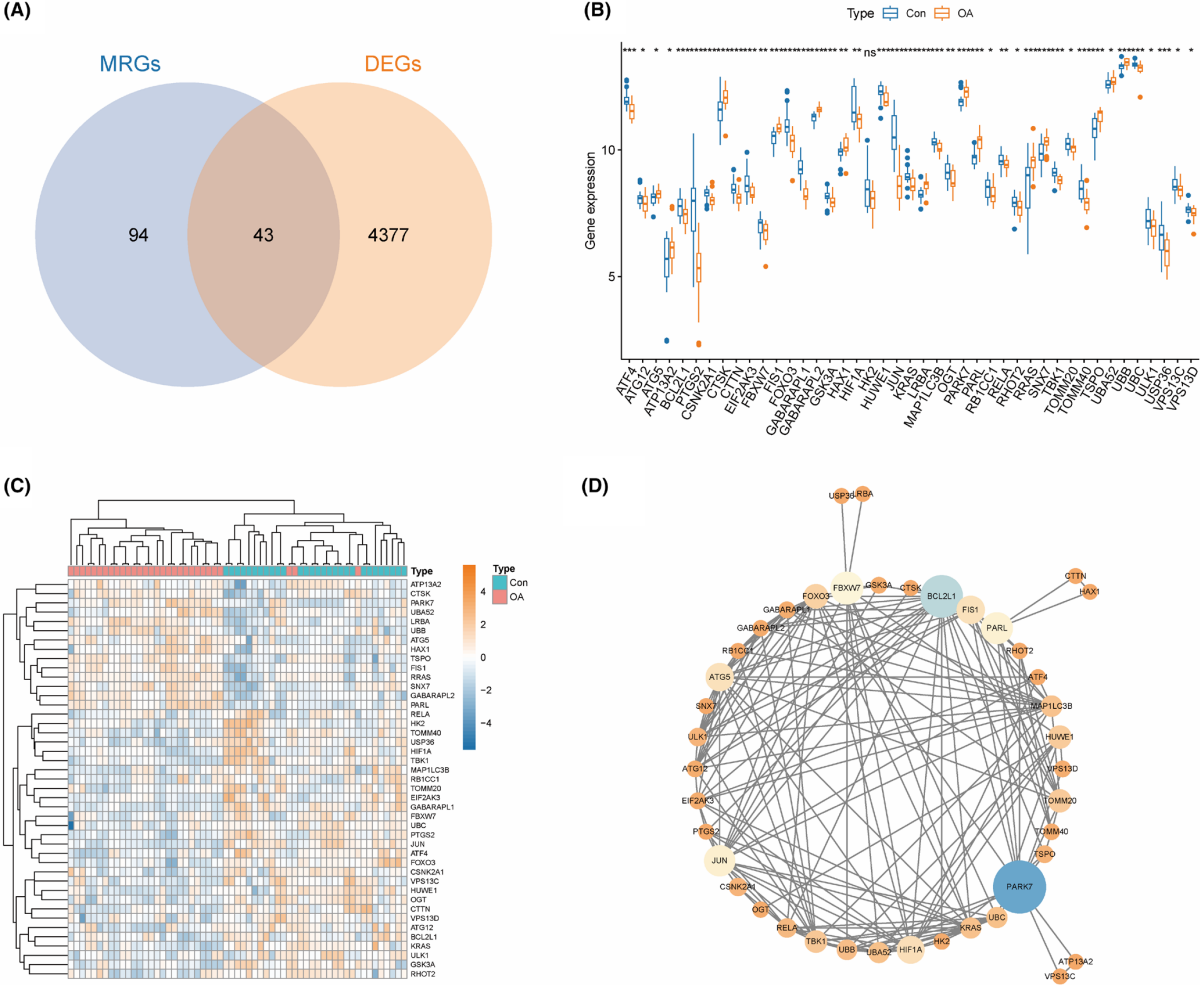
The expression of DEMRGs in OA and their PPI network. (A), The Venn diagram of the intersection of DEGs and MRGs, where the intersection part is called DEMRGs. (B), The expression boxplot of DEMRGs in OA‐related training datasets. (C), Expression heatmap of DEMRGs in OA‐related training datasets. Yellow represents genes upregulated in OA, while blue represents genes downregulated in OA. Red represents OA samples, while blue represents normal samples. (D), 42 DEMRGs' PPI network. From yellow to blue, the darker the color, the larger the node, the greater the degree and importance of the gene in the network.

To identify DEMRGs in OA, we intersected 137 MRGs with 4420 DEGs from the OA‐related merged dataset, resulting in a total of 43 DEMRGs (Figure 2A). More than half of the 43 DEMRGs were downregulated in OA, with 14 DEMRGs upregulated and 29 DEMRGs downregulated (Figure 2B,C). PPI analysis showed that a total of 42 genes in 43 DEMRGs had protein interaction relationships. We inputted the interaction network of 42 DEMRGs into Cytoscape. Then, based on the degree calculated by the Analyze Network plugin, the importance of each DEMRG in the PPI network is visualized (Figure 2D). We found that PARK7, BCL2L1, FBXW7, HIF1A, JUN, and ATG5 have a greater degree in the PPI network, which also means that their importance in the PPI network is higher.

**FIGURE 3:**
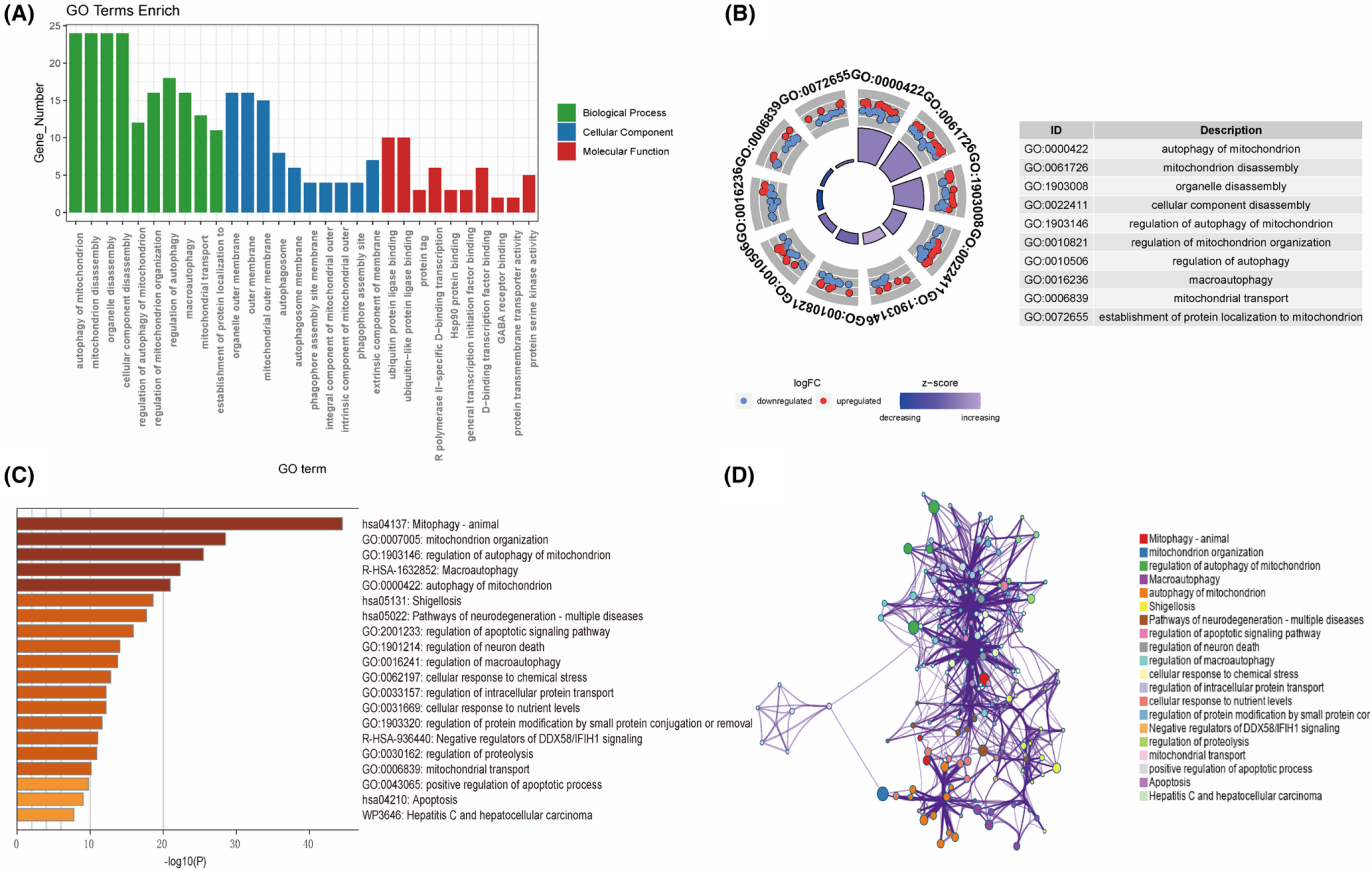
Functional enrichment analysis of 43 DEMRGs in OA. (A,B), GO analysis results of 43 DEMRGs. (C,D), Metascape analysis results of 43 DEMRGs.

Furthermore, we conducted GO and Metascape analyses on 43 DEMRGs to elucidate their functions and pathways in OA enrichment. The GO analysis results indicate that 43 DEMRGs are mainly involved in functional pathways such as autophagy of mitochondrion, mitochondrion disassembly, organelle disassembly, and cellular component disassembly (Figure 3A,B). The Metascape analysis results indicated that 43 DEMRGs are mainly involved in signal pathways such as Mitophagy animal, Mitochondrion organization, Regulation of autophagy of mitochondrion, Macroautophagy, and Pathways of Neurogeneration multiple diseases (Figure 3C,D).

### 3.3 The diagnostic value of MRGs in OA

**FIGURE 4:**
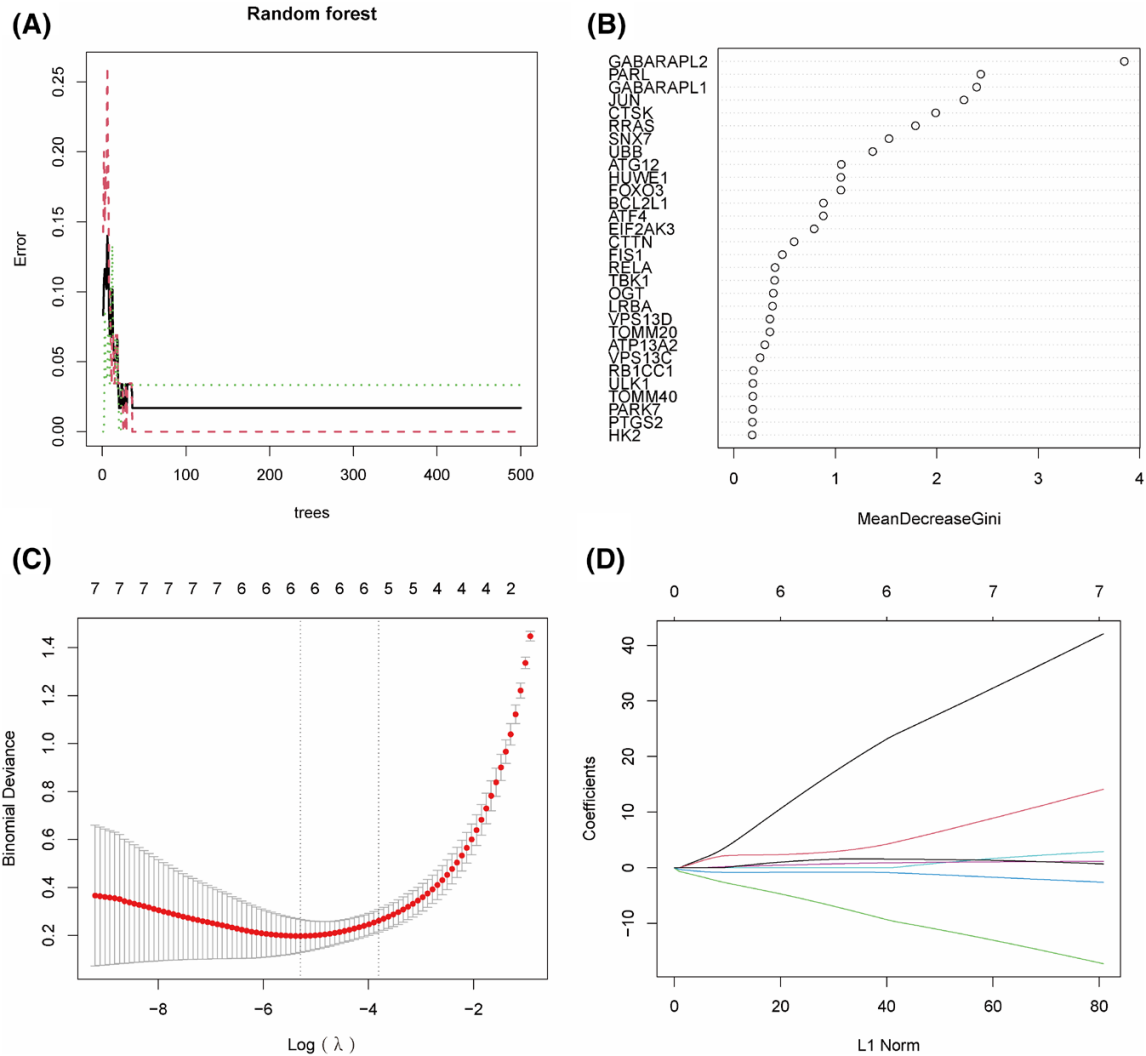
Identification of diagnostic genes in OA. (A), The error rate of RF under different option trees (1–500). (B), Top 30 important rating rankings for DEMRGs. (C), Cross validation to select the optimal tuning parameter log(λ). (D), LASSO coefficient profiles of candidate genes.

**FIGURE 5:**
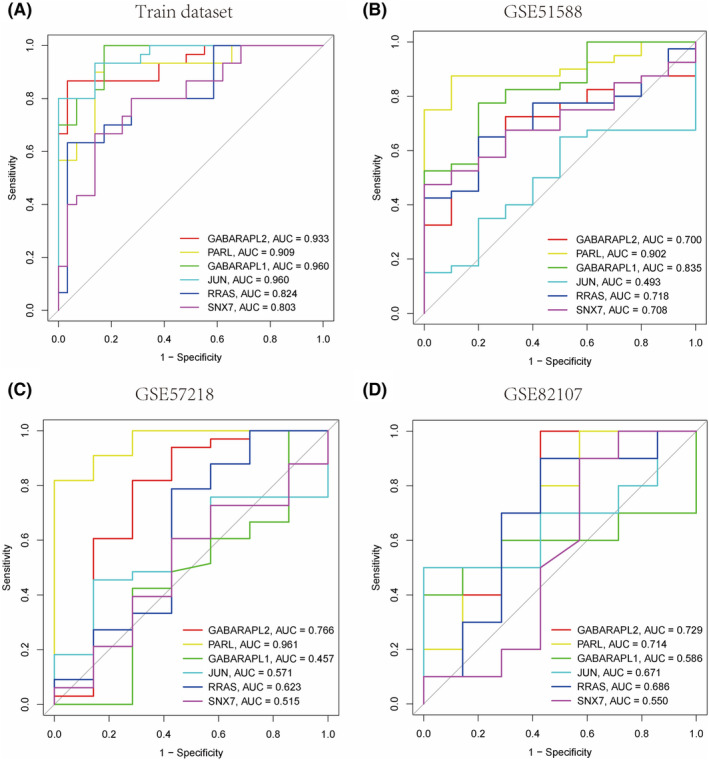
ROC analysis. Receiver operating characteristic of 6 diagnostic‐related DEMRGs in OA‐related training dataset (A), GSE51588 (B), GSE82107 (C) and GSE57218 (D).

To evaluate the diagnostic value of DEMRGs in OA, based on the training dataset related to OA, we first used RF to calculate the importance of 43 DEMRGs. In Figure 4A, the error rates corresponding to option trees from 1 to 500 are shown, and the error is minimized when option trees are 20. Then, we ranked the importance of the top 30 DEMRGs and retained genes with an importance score greater than 1.5, with a total of 7 DEMRGs (GABARAPL2, PARL, GABARAPL1, JUN, CTSK, RRAS, and SNX7) as inputs to the LASSO algorithm (Figure 4B). LASSO analysis is used to further identify MRGs‐related to OA diagnosis. The results showed that LASSO analysis identified 6 diagnostic genes, including GABARAPL2, PARL, GABARAPL1, JUN, RRAS, and SNX7 (Figure 4C,D). To evaluate the diagnostic accuracy of these genes, we conducted ROC analysis on the OA‐related training dataset. In Figure 5A, GABARAPL2 (AUC = 0.933), PARL (AUC = 0.909), GABARAPL1 (AUC = 0.960), JUN (AUC = 0.960), RRAS (AUC = 0.824), and SNX7 (AUC = 0.803) showed high accuracy in the diagnosis of OA. The GSE51588, GSE82107, and GSE57218 datasets were used to further validate the diagnostic efficacy of these diagnostic genes. The results showed that these diagnostic genes were able to diagnose OA with medium to high accuracy in the GSE51588 (Figure 5B), GSE82107 (Figure 5C), and GSE57218 datasets (Figure 5D). The above results indicate that GABARAPL2, PARL, GABARAPL1, JUN, RRAS, and SNX7 have high diagnostic value in OA.

### 3.4 Clinical utility of diagnostic‐related MRGs in OA

**FIGURE 6:**
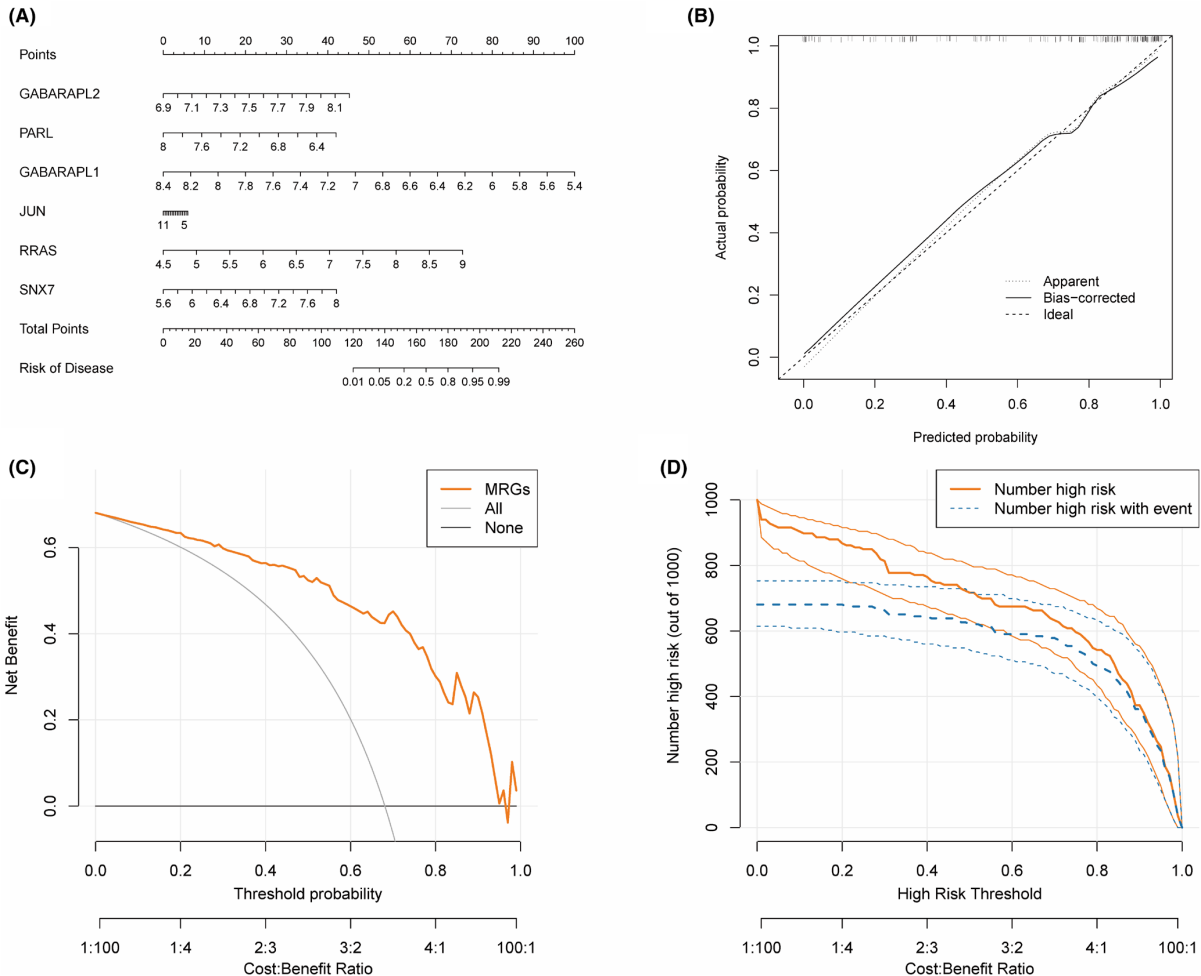
Construction of MRG‐related nomogram. (A), Nomogram. (B), Calibration curve constructed by 6 diagnostic‐related MRGs. The x‐axis represents the diagnostic accuracy probability of the nomogram, while the y‐axis represents the actual prediction accuracy. ‘Ideal’ represents the ideal predicted value. The dashed line corresponding to ‘Apparent’ represents the predicted value of the uncorrected nomogram, while the dashed line corresponding to ‘Bias‐corrected’ represents the predicted value of the corrected nomogram. (C), Decision tree curve. The yellow line represents the net income of the nomogram model, the gray line represents the assumption that all samples are OA, and the black horizontal line represents the assumption that there are no OA patients. (D), Clinical impact curve. The yellow curve represents the predicted number of OA patients with different threshold probabilities, while the blue curve represents the actual number of OA patients.

Based on 6 diagnostic‐related MRGs (GABARAPL2, PARL, GABARAPL1, JUN, RRAS, and SNX7), a nomogram was constructed using the ‘rms’ package to further estimate the clinical guidance value of MRGs in OA. In Figure 6A, OA patients can calculate the corresponding disease risk probability values based on the total scores of 6 diagnostic‐related MRGs. The calibration curve shows that the performance of the nomogram model is similar to that of the ideal model (Figure 6B). In addition, the DCA analysis results showed that if the threshold probability is less than 0.98, using this nomogram to predict the probability of disease improvement will result in more net benefits than schemes with all or no OA patients. (Figure 6C). The clinical impact curve showed that when the threshold probability is greater than 0.2, the number of predicted patients is close to the actual number of OA patients, and the cost–benefit ratio is 0.25 (Figure 6D). The above analysis results indicated that the nomogram constructed based on 6 diagnostic‐related MRGs has high clinical application potential.

### 3.5 Correlation between MRGs and immune microenvironment in OA

**FIGURE 7:**
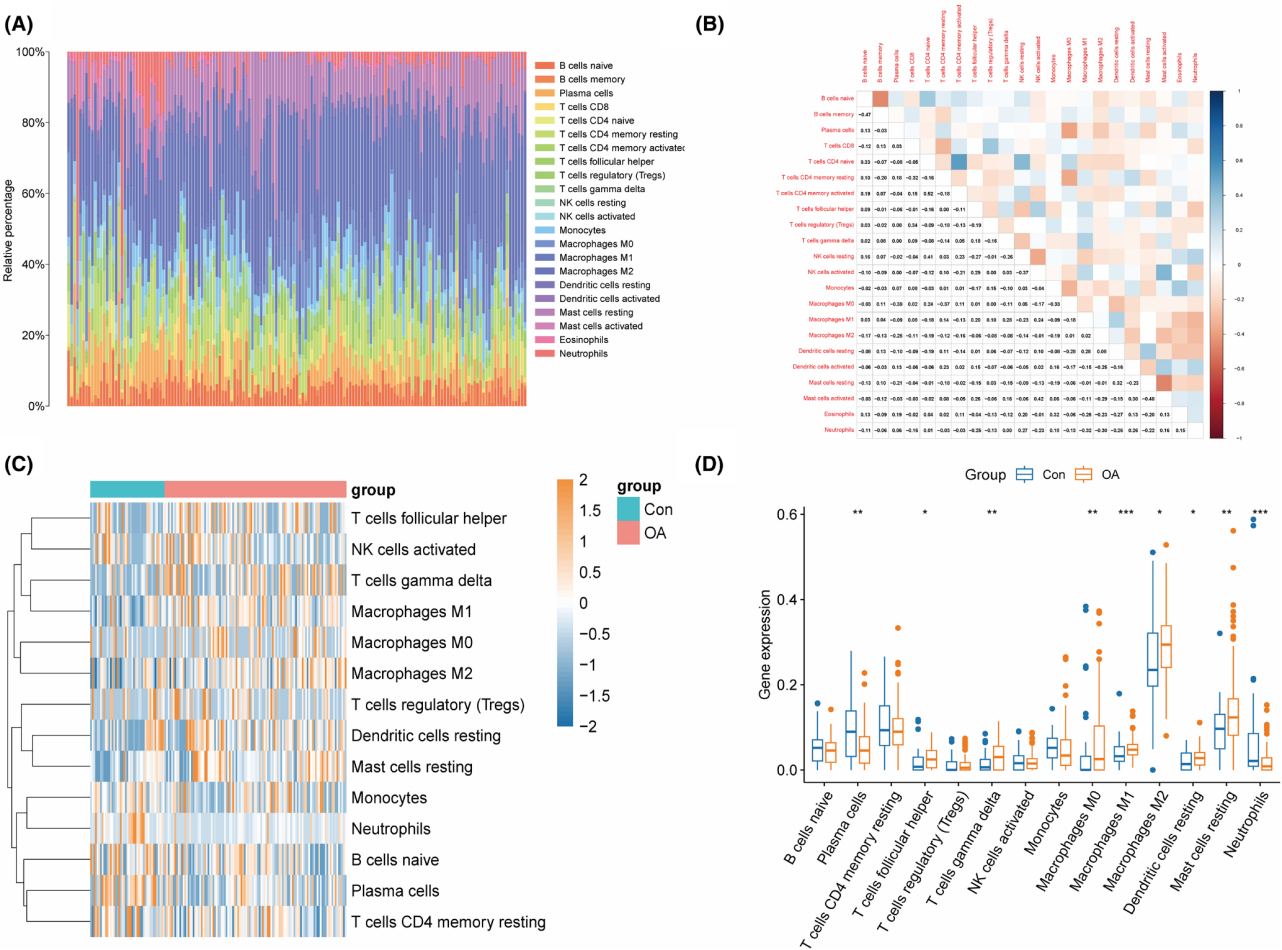
Immune differences between OA and normal samples. (A), Barplot showed the proportion of 22 immune cells between OA and normal samples. (B), 22 types of immune cell correlation heatmaps. (C), Heatmap of abundance levels of different immune cells (abundance greater than 0 in over half of the samples) between OA and normal samples. (D), Boxplot of abundance differences between OA and normal samples for different immune cells (abundance greater than 0 in over half of the samples).

Based on the OA‐related merged dataset (training dataset, GSE51588, GSE82107, and GSE57218), the immune infiltration abundance of 22 immune cells in OA and normal samples was calculated using CIBERSORT analysis. We found that Macrophages, T cells, and Mast cells are more commonly present in OA (Figure 7A). The correlation analysis between immune cells showed that there are extensive connections between different immune cells in OA (Figure 7B). T cells CD4 memory activated and T cells CD4 memory resetting (cor = 0.52), Mast cells activated and NK cells activated (cor = 0.42), NK cells resetting and T cells CD4 naive (cor = 0.41) have a high positive correlation, while Mast cells activated and Mast cells resetting (cor = −0.48), B cells memory and B cells naive (cor = −0.47) have a high negative correlation. We visualized the abundance of immune cells (with an abundance greater than 0 in more than half of the samples) in OA and normal samples through heatmaps (Figure 7C). In order to compare the differences in immune cell infiltration between OA and normal samples more intuitively, we used wilcox.test to calculate the *p* value of the abundance difference between different immune cells (with an abundance greater than 0 in more than half of the samples). We found that the abundance of most differentially expressed immune cells was upregulated in OA, including T cells follicular helper (*p* < 0.05), T cells gamma delta (*p* < 0.01), Macrophages M0 (*p* < 0.01), Macrophages M1 (*p* < 0.001), Macrophages M2 (*p* < 0.05), Dendritic cells resetting (*p* < 0.05), and Mast cells resetting (*p* < 0.01), while the abundance levels of Plasma cells (*p* < 0.01) and Neutrophils (*p* < 0.001) were downregulated in OA (Figure 7D). The results indicated that the progression of OA may be‐related to a decrease in immune levels.

**FIGURE 8:**
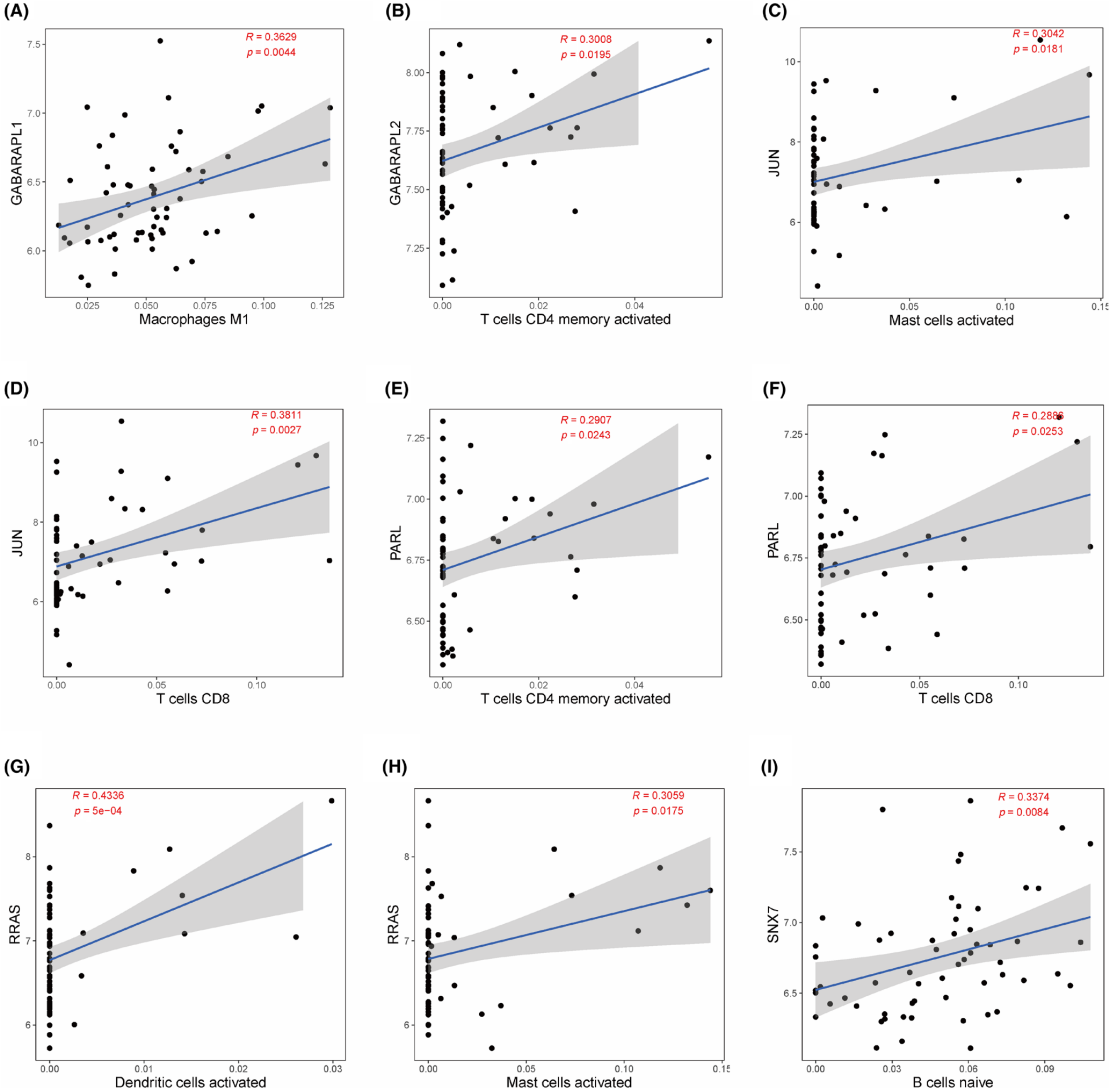
Analysis of the correlation between diagnostic‐related MRGs and immune cells. GABARAPL1 was significantly correlated with Macrophages M1 (A), T cells CD4 memory activated (B), JUN was significantly correlated with Mast cells activated (C), T cells CD8 (D), PARL was significantly correlated with T cells CD4 memory activated (E), T cells CD8 (F), RRAS was significantly correlated with Dendritic cells activated (G), Mast cells activated (H), SNX7 and B cells naive (I).

To further determine the impact of MRGs in the OA immune microenvironment, we used Pearson's method to calculate the correlation between 6 diagnostic‐related MRGs (GABARAPL2, PARL, GABARAPL1, JUN, RRAS, and SNX7) and 22 immune cells. The results showed that GABARAPL1 was significantly correlated with Macrophages M1 (*R* = 0.3629, *p* = 0.0044) and T cells CD4 memory activated (*R* = 0.3008, *p* = 0.0195), JUN was significantly correlated with Mast cells activated (*R* = 0.3042, *p* = 0.0181) and T cells CD8 (*R* = 0.3811, *p* = 0.0027), PARL was significantly correlated with T cells CD4 memory activated (*R* = 0.2907, *p* = 0.0243) and T cells CD8 (*R* = 0.2886, *p* = 0.0253), RRAS was significantly correlated with Dendritic cells activated (*R* = 0.4336, *p* = 5e‐04) and Mast cells activated (*R* = 0.3059, *p* = 0.0175), while SNX7 and B cells naive (*R* = 0.3374, *p* = 0.0084) were significantly correlated (Figure 8A–I). Our results suggested that 6 diagnostic‐related MRGs may be associated with changes in immune cell infiltration levels in OA.

### 3.6 Verification of the expression of diagnostic‐related MRGs

**FIGURE 9:**
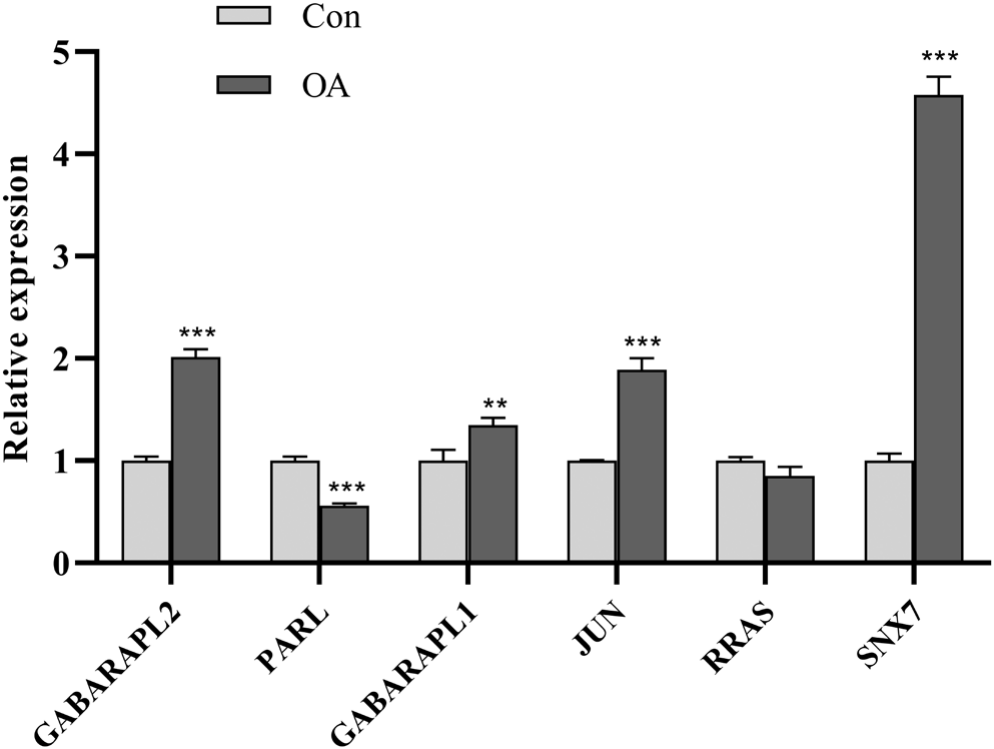
The relative mRNA expression of the diagnostic gene (GABARAPL2, PARL, GABARAPL1, JUN, RRAS, and SNX7) in OA was analyzed using qRT‐PCR. **p < 0.01, and ***p < 0.001.

We used qRT‐PCR to analyze the relative mRNA expression of diagnostic‐related genes in OA. The experimental results showed that compared with normal tissues, the expression of GABARAPL2, GABARAPL1, JUN, and SNX7 were upregulated in OA, while the expression of PARL and RRAS were downregulated (Figure 9).

## 4 DISCUSSION

The diagnosis and treatment of OA face challenges, and there is an urgent need to develop new diagnostic methods and treatment strategies. More and more evidence has suggested that mitophagy plays an important role in the pathogenesis and immune response regulation of OA. However, no research has elucidated the molecular mechanism, clinical value, and role of mitophagy in OA immune system.

In this study, 4420 DEGs were identified in OA through differential analysis. The GSEA analysis results show that DEGs in OA are mainly enriched in pathways such as Allograft rejection, Apoptosis, Insulin signaling pathway, JAK STAT signaling pathway, Lysosome, MAPK signaling pathway, Oxidative phonology, Parkinsons disease, and T cell receiver signaling pathway. Allograft rejection plays an important role in the treatment of OA.^19^ Apoptosis obviously occurs in the cartilage of OA, and the imbalance of apoptosis may lead to the pathological changes of OA.^20^ JAK STAT signaling pathway is activated by inflammatory factors and affects the pathophysiological activity of chondrocytes in OA by regulating cell proliferation, differentiation, and apoptosis.^21^ Previous studies found that fargesin reprogrammed macrophages by downregulating MAPK signaling pathway and improved the pathological progress of OA.^22^ The research of Jin et al. confirmed that curcumin plays a cartilage protective role on OA by activating mitophagy mediated by Parkinsons disease.^23^ In addition, the insulin signaling pathway and oxidative physiology are also associated with mitophagy.^24^, ^25^ The above results indicated that these differential genes may play an important role in the pathological changes of OA.

Subsequently, we intersected 4420 DEGs with 137 MRGs and obtained 43 DEMRGs. Our results showed that over half of DEMRGs were downregulated in OA. The enrichment analysis results showed that the above 43 DEMRGs were mainly involved in mitophagy and degenerative disease‐related pathways, including Mitophagy animal, Mitochondrion organization, Regulation of autophagy of mitochondrion, Macroautophagy, and Pathways of neurogeneration multiple diseases. PPI analysis showed that PARK7, BCL2L1, FBXW7, HIF1A, JUN, and ATG5 are of high importance in the interaction network.

To determine the diagnostic and clinical value of MRGs in OA, we first identified 6 MRGs related to OA diagnosis using RF and LASSO algorithms: GABARAPL2, PARL, GABARAPL1, JUN, RRAS, and SNX7. ROC analysis showed that the 6 diagnostic‐related MRGs mentioned above can predict OA and normal samples with high accuracy. Among them, GABARAPL1 (AUC = 0.960) and JUN (AUC = 0.960) have the highest AUC values and higher diagnostic accuracy. In addition, the above 6 diagnostic‐related MRGs also demonstrated good diagnostic performance in the validation dataset. Given the diagnostic value of the 6 diagnostic‐related MRGs mentioned above, we conducted a column chart analysis. The calibration curve analysis shows that the predictive performance of the nomogram model based on 6 diagnostic‐related MRGs is similar to that of the ideal model. The results of DCA analysis and clinical impact curve analysis indicated that nomograms have great potential in the clinical application of OA.

Immunity plays a stimulating and regulatory role in the progression of OA.^26^ The CIBERSORT analysis results indicated that macrophages, T cells, and mast cells are more commonly present in OA, and there are extensive interactions between different immune cells. Furthermore, we found that the abundance of most significantly different immune cells was upregulated in OA, including T cell follicular helper, T cell gamma delta, Macrophages M0, Macrophages M1, Macrophages M2, Dendritic cells resetting, Mast cells resetting, while the abundance levels of Plasma cells and Neutrophils were downregulated in OA. Under different pathological conditions, activated immune cells such as T lymphocytes, macrophages and dendritic cells produce a large number of proinflammatory cytokines, and affect the activity of Osteoclast and Bone resorption.^27^ Research has found that activation of the immune system leads to increased inflammation and joint damage.^16^ Therefore, regulating these immune cells may play an important role in alleviating the pathological progression of OA patients. In addition, our study suggested that MRGs are associated with immune cells in OA, such as GABARAPL1 being significantly correlated with Macrophages M1, T cells CD4 memory activated, and JUN being significantly correlated with Mast cells activated and T cells CD8. The above results indicate that mitophagy plays a regulatory role in the immune microenvironment of OA.

Finally, we identified GABARAPL2, PARL, GABARAPL1, JUN, RRAS, and SNX7 as diagnostic biomarkers for OA. The results of bioinformatics analysis and qPCR experiments showed that GABARAPL2, PARL, RRAS, and SNX7 were upregulated in OA, while GABARAPL1 and JUN were downregulated in OA. GABARAPL2 prevents excessive ROS production by regulating mitochondrial levels.^28^ The excessive production of ROS will change the life cycle and metabolism of chondrocytes, and lead to synovitis and cartilage dysfunction, which is one of the causes of OA.^29^ Zhang et al. identified GABARAPL2 as a biomarker related to autophagy in OA, and RT‐PCR analysis showed that the expression of GABARAPL2 in OA was upregulated.^30^ GABARAPL1 plays a key role in the maturation of autophagosome.^31^ According to reports, inhibiting autophagy in OA exacerbates the degeneration of chondrocytes and cartilage.^32^ Therefore, GABARAPL1 may affect the pathological changes of OA by regulating autophagy in OA. The protein encoded by PARL regulates mitochondrial remodeling by regulating substrate protein hydrolysis.^33^ Mitochondrial remodeling is a key process in maintaining cellular homeostasis and plays an important role in regulating skeletal muscle regeneration.^34^ Li et al. improved the degree of cartilage damage in OA by inhibiting the transcriptional activity of JUN in the OA mouse model.^35^ RRAS is a small GTPase that plays a regulatory role in actin cytoskeleton and cell adhesion.^36^, ^37^ In the pathogenesis of OA, cell adhesion molecules can recruit monocytes in inflamed synovium tissue, thus leading to the deterioration of OA.^38^ SNX7 interacts with SNX4 to participate in autophagosome assembly by regulating the transport and recovery of ATG9A.^39^ Pathological cartilage calcification occurs in the early stage of OA, and autophagosome carrying minerals is the key factor to induce calcification.^40^

In summary, our study demonstrates that six MRGs (GABARAPL2, PARL, GABARAPL1, JUN, RRAS, and SNX7) can be used as diagnostic biomarkers with high predictive accuracy, which may be helpful in the diagnosis of OA. The expression of diagnostic‐related MRGs is correlated with changes in the abundance of immune cells in OA. At the same time, mitophagy may affect the immune microenvironment of OA by regulating immune cells, ultimately leading to the progression of OA. The results of this study provide insights into the pathogenesis and progression of OA, as well as suggesting promising targets for the diagnosis and treatment of OA.

## AUTHOR CONTRIBUTIONS

D.X. Tang and H. Song: Data curation, Formal analysis, Writing‐original draft, Software. Y.F. Luo: Re‐sources, Formal analysis. S.Q. Ruan: Writing‐review and editing, Resources, Conceptualization, Funding acquisition, Project administration. All authors have read and agreed to the published version of the manuscript.

## FUNDING INFORMATION

This work was supported by Zunyi Science and Technology Bureau and the First People's Hospital of Zunyi joint science and Technology Research and development fund project (No. Zun City Science and HZ word (2023) 16); Guizhou Province Science and Technology Program Project (No. Guizhou Science and Technology‐ZK [2021] General 393); Guizhou Provincial Health Commission Science and Technology Foundation Project (No. gzwkj2021‐259); Guizhou Province Science and Technology Program Project (No. Guizhou Synthetic Fruit‐LC [2022]029).

## CONFLICT OF INTEREST STATEMENT

The authors confirm that there are no conflicts of interest.

## ETHICS APPROVAL AND CONSENT TO PARTICIPATE

All patients gave consent for publication. This study was approved by the Institutional Review Board of the First People‘s Hospital of Zunyi on September 26, 2018 (local ethics committee reference number 2020086).

## Supporting information


**Data S1.** Supporting Information.
