# Supplementary figures and images for: Identification of mitophagy‐related biomarkers in osteoarthritis
Source: Animal Model Exp Med. 2024 May 8;7(6):781–92. doi: 10.1002/ame2.12416 (PMC11680475; doi:10.1002/ame2.12416)

# KEGG\_ADIPOCYTOKINE\_SIGNALING\_PATHWAY

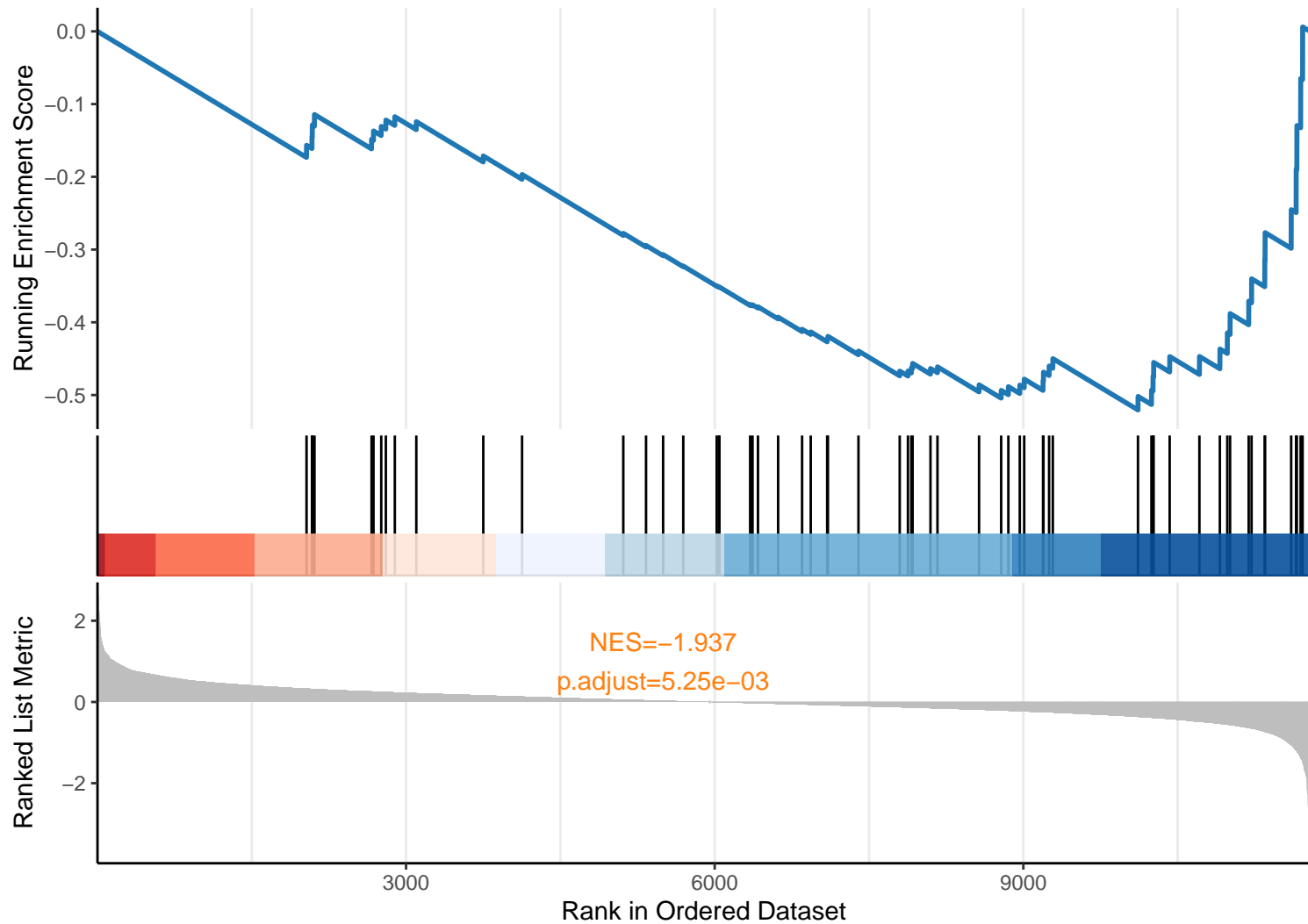

Supplement: Supplementary file 1 — Data S1. Supporting Information. [file AME2-7-781-s001.zip › Supplementary Material 4/KEGG_ADIPOCYTOKINE_SIGNALING_PATHWAY_gseaplot.pdf]

# KEGG\_CYTOKINE\_CYTOKINE\_RECEPTOR\_INTERACTION

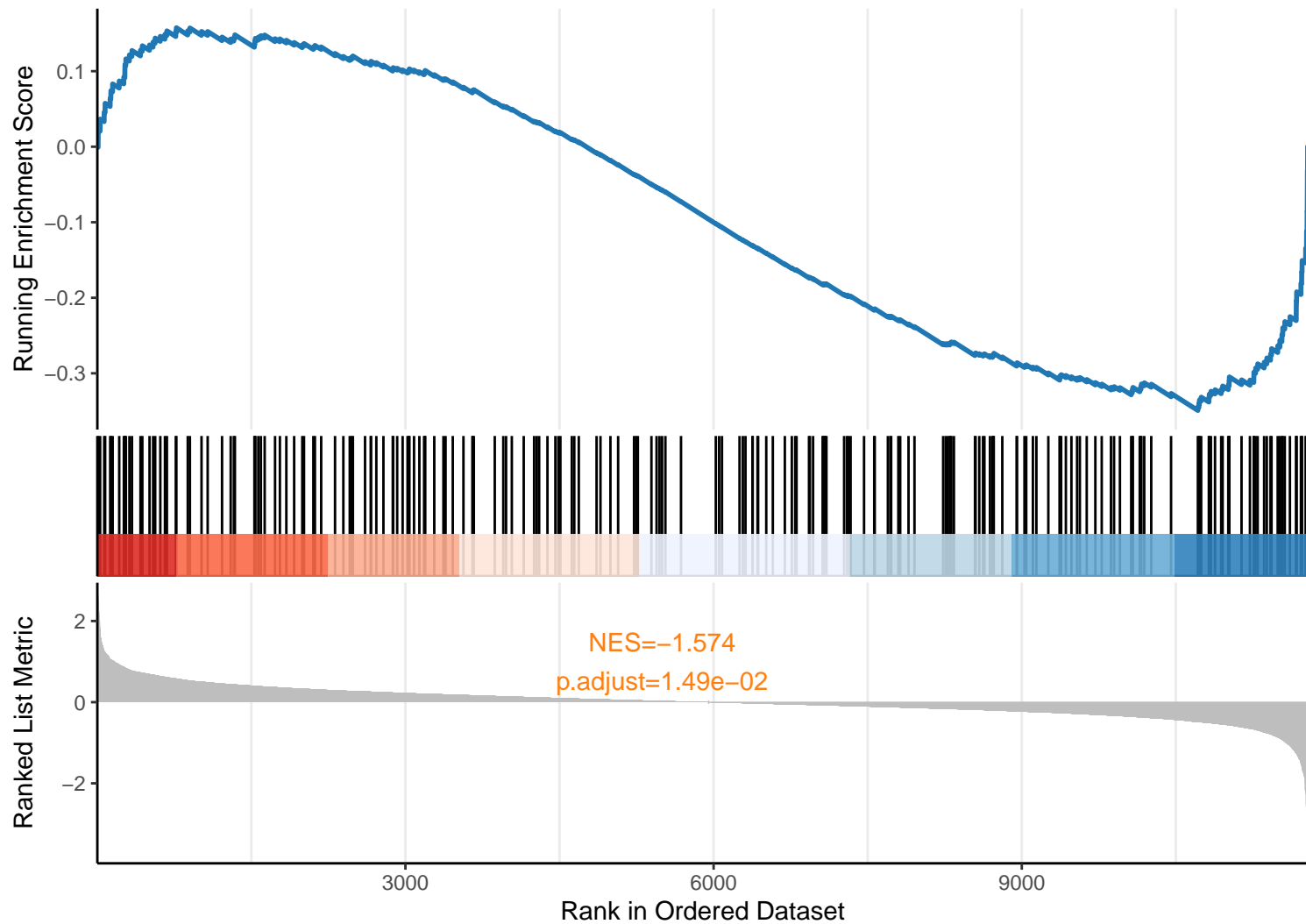

Supplement: Supplementary file 1 — Data S1. Supporting Information. [file AME2-7-781-s001.zip › Supplementary Material 4/KEGG_CYTOKINE_CYTOKINE_RECEPTOR_INTERACTION_gseaplot.pdf]

# KEGG\_ERBB\_SIGNALING\_PATHWAY

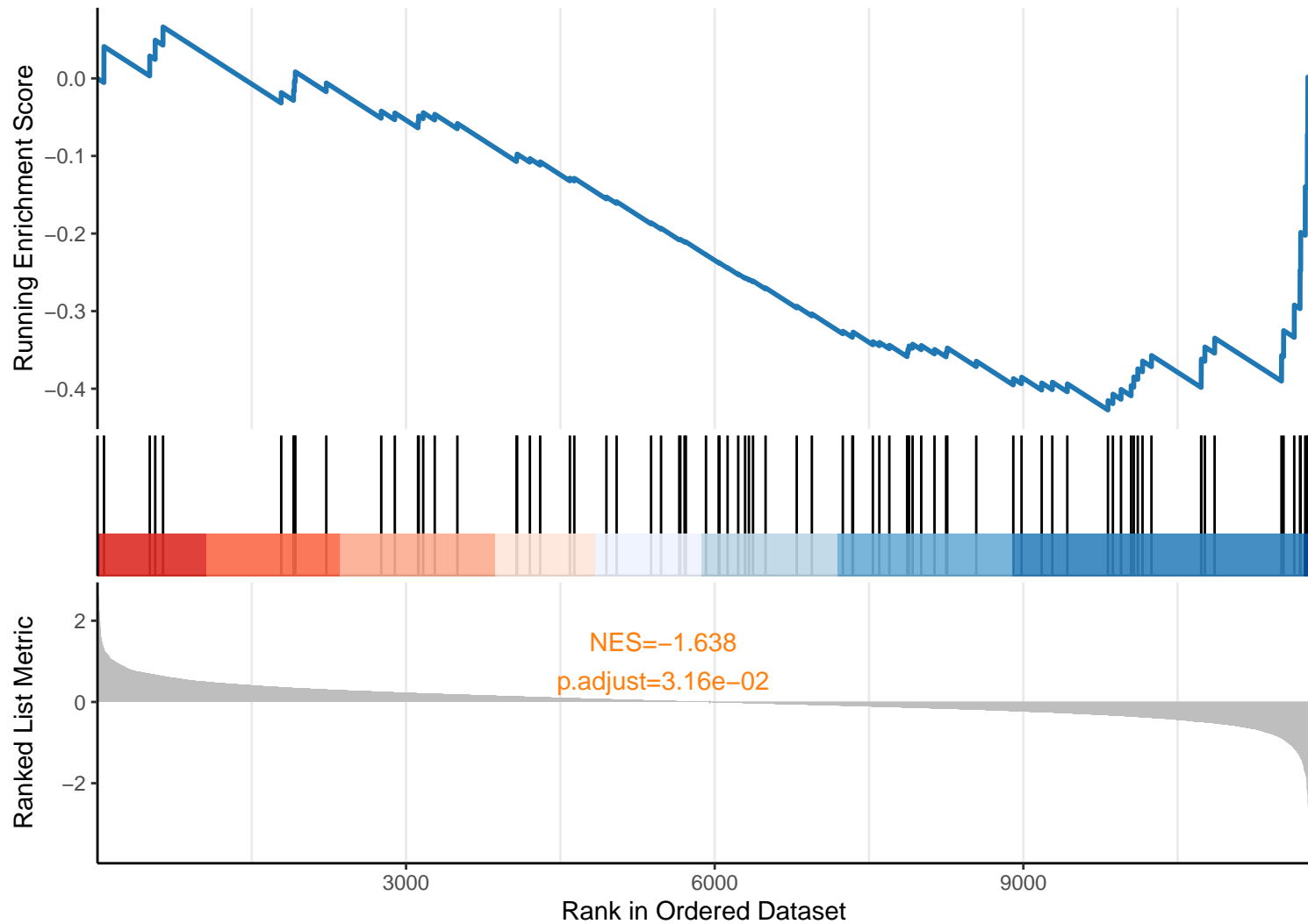

Supplement: Supplementary file 1 — Data S1. Supporting Information. [file AME2-7-781-s001.zip › Supplementary Material 4/KEGG_ERBB_SIGNALING_PATHWAY_gseaplot.pdf]

# KEGG\_NOD\_LIKE\_RECEPTOR\_SIGNALING\_PATHWAY

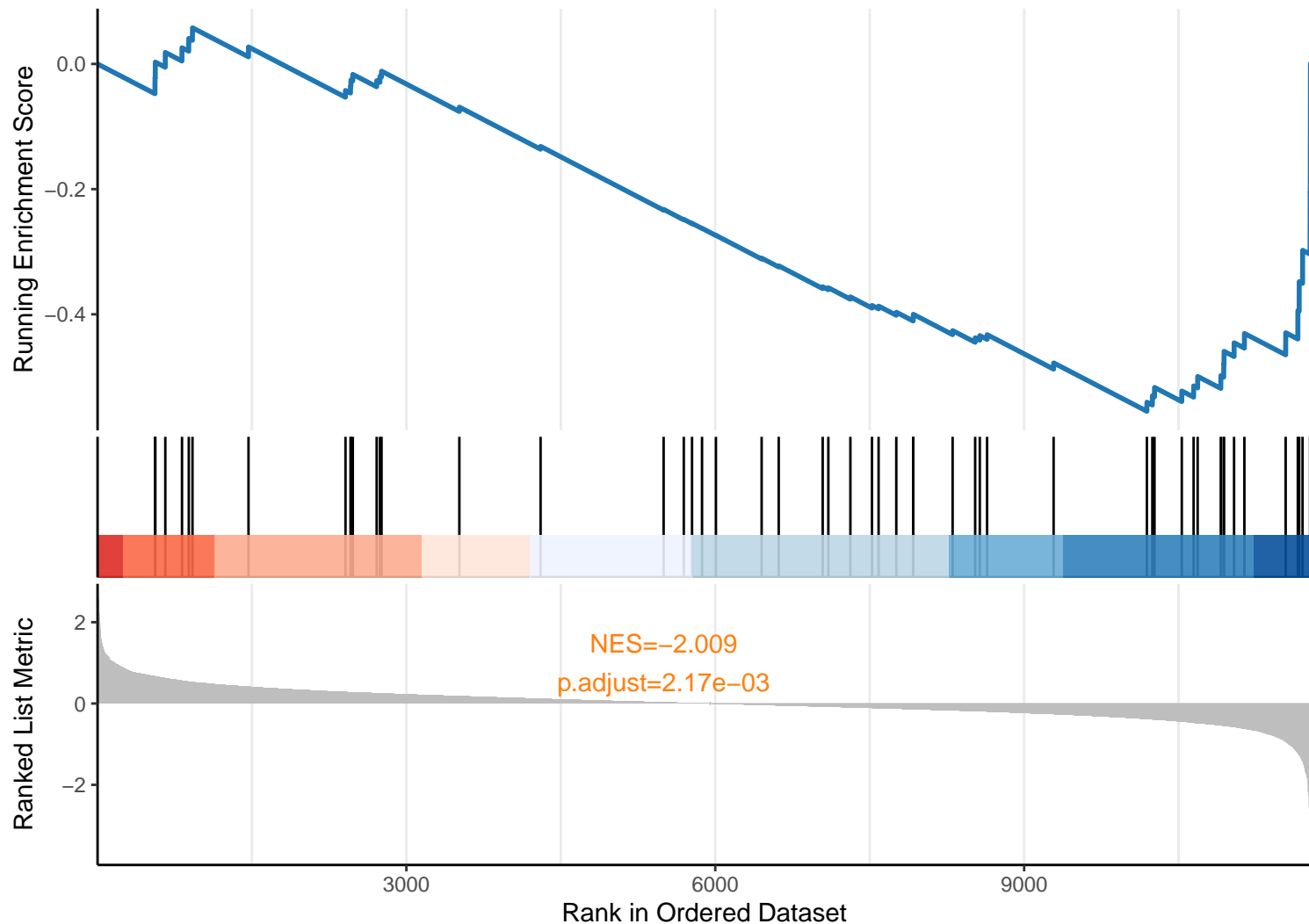

Supplement: Supplementary file 1 — Data S1. Supporting Information. [file AME2-7-781-s001.zip › Supplementary Material 4/KEGG_NOD_LIKE_RECEPTOR_SIGNALING_PATHWAY_gseaplot.pdf]

# KEGG\_PATHWAYS\_IN\_CANCER

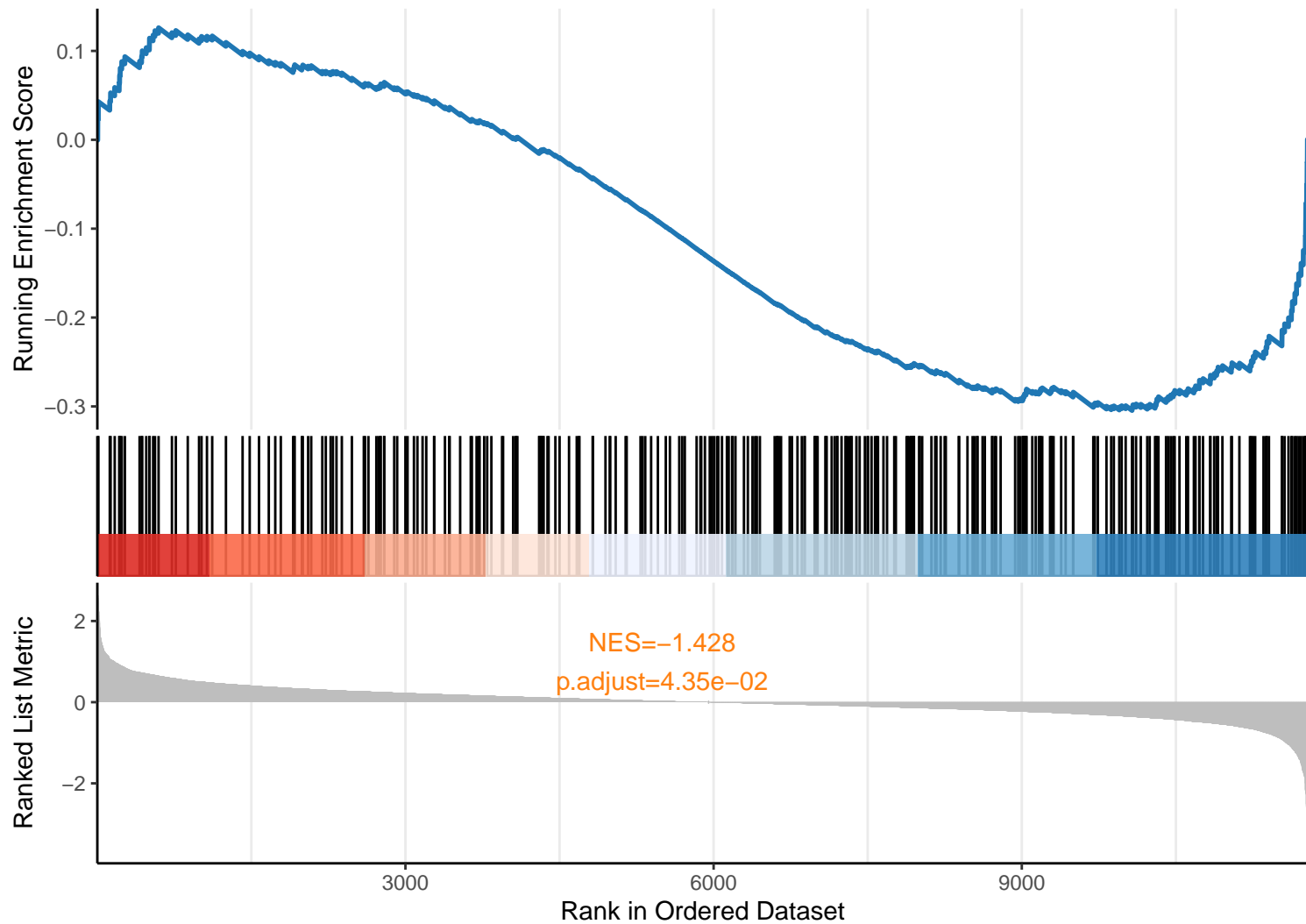

Supplement: Supplementary file 1 — Data S1. Supporting Information. [file AME2-7-781-s001.zip › Supplementary Material 4/KEGG_PATHWAYS_IN_CANCER_gseaplot.pdf]

# KEGG\_PEROXISOME

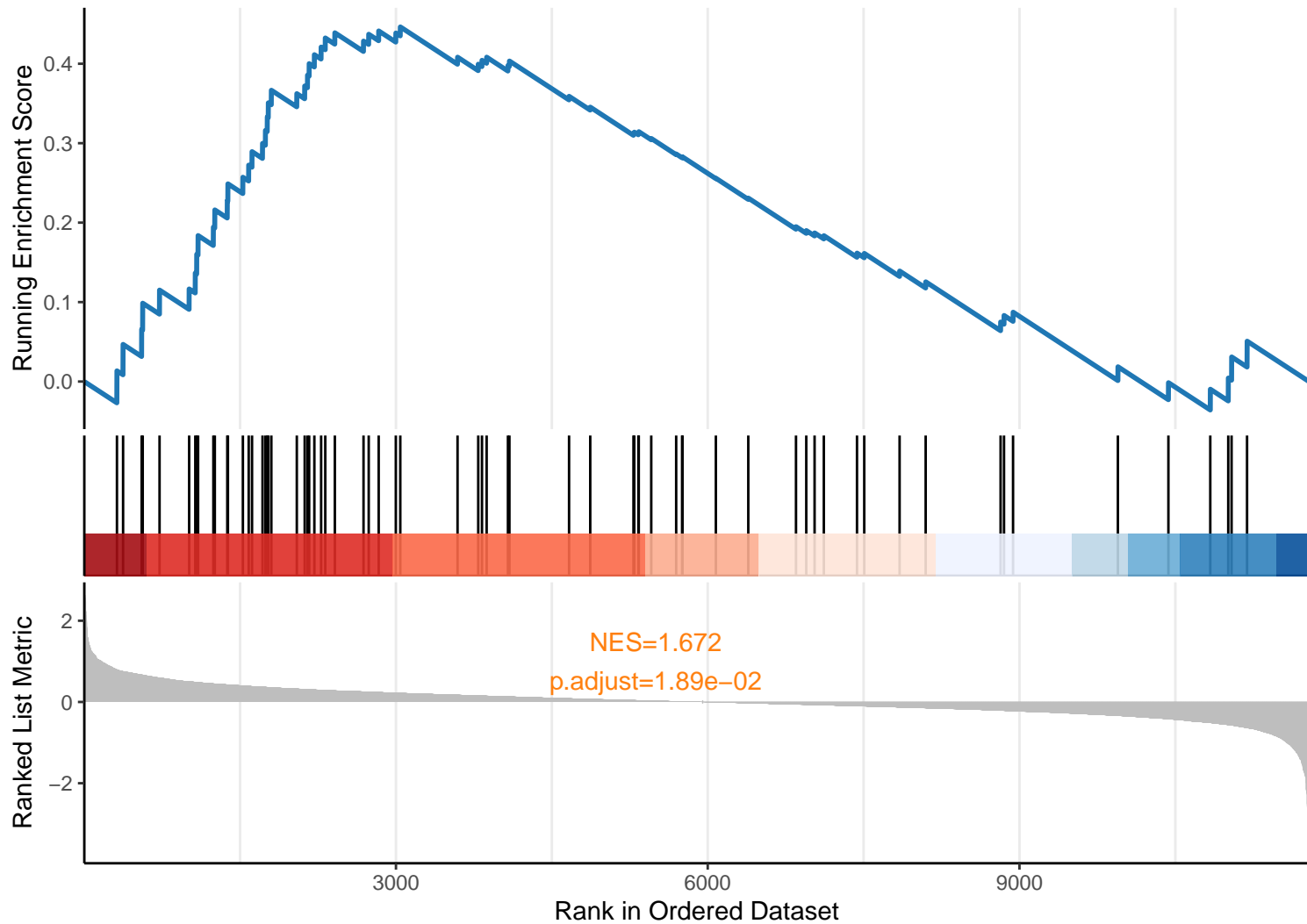

Supplement: Supplementary file 1 — Data S1. Supporting Information. [file AME2-7-781-s001.zip › Supplementary Material 4/KEGG_PEROXISOME_gseaplot.pdf]

# KEGG\_SMALL\_CELL\_LUNG\_CANCER

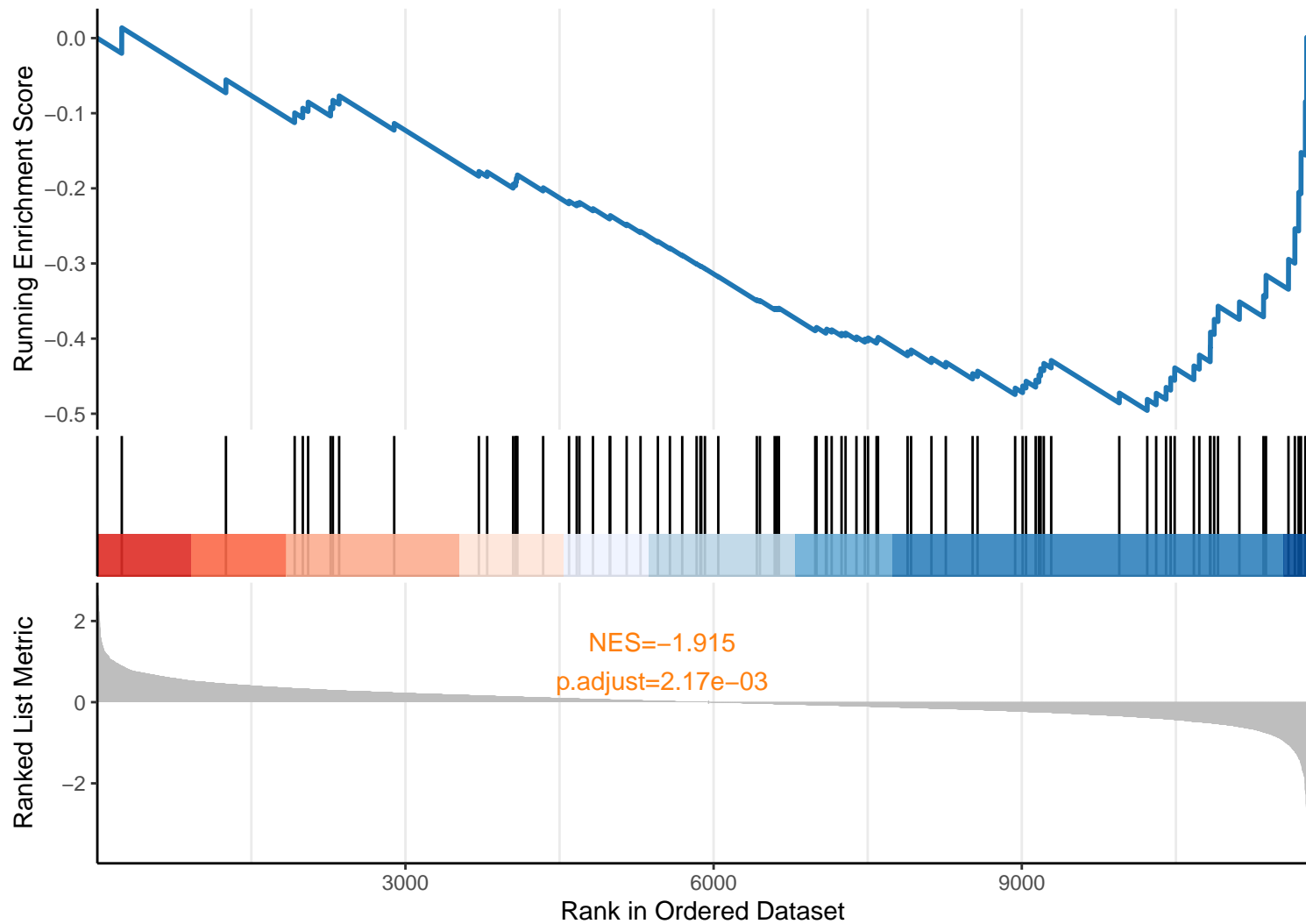

Supplement: Supplementary file 1 — Data S1. Supporting Information. [file AME2-7-781-s001.zip › Supplementary Material 4/KEGG_SMALL_CELL_LUNG_CANCER_gseaplot.pdf]

# KEGG\_SPLICEOSOME

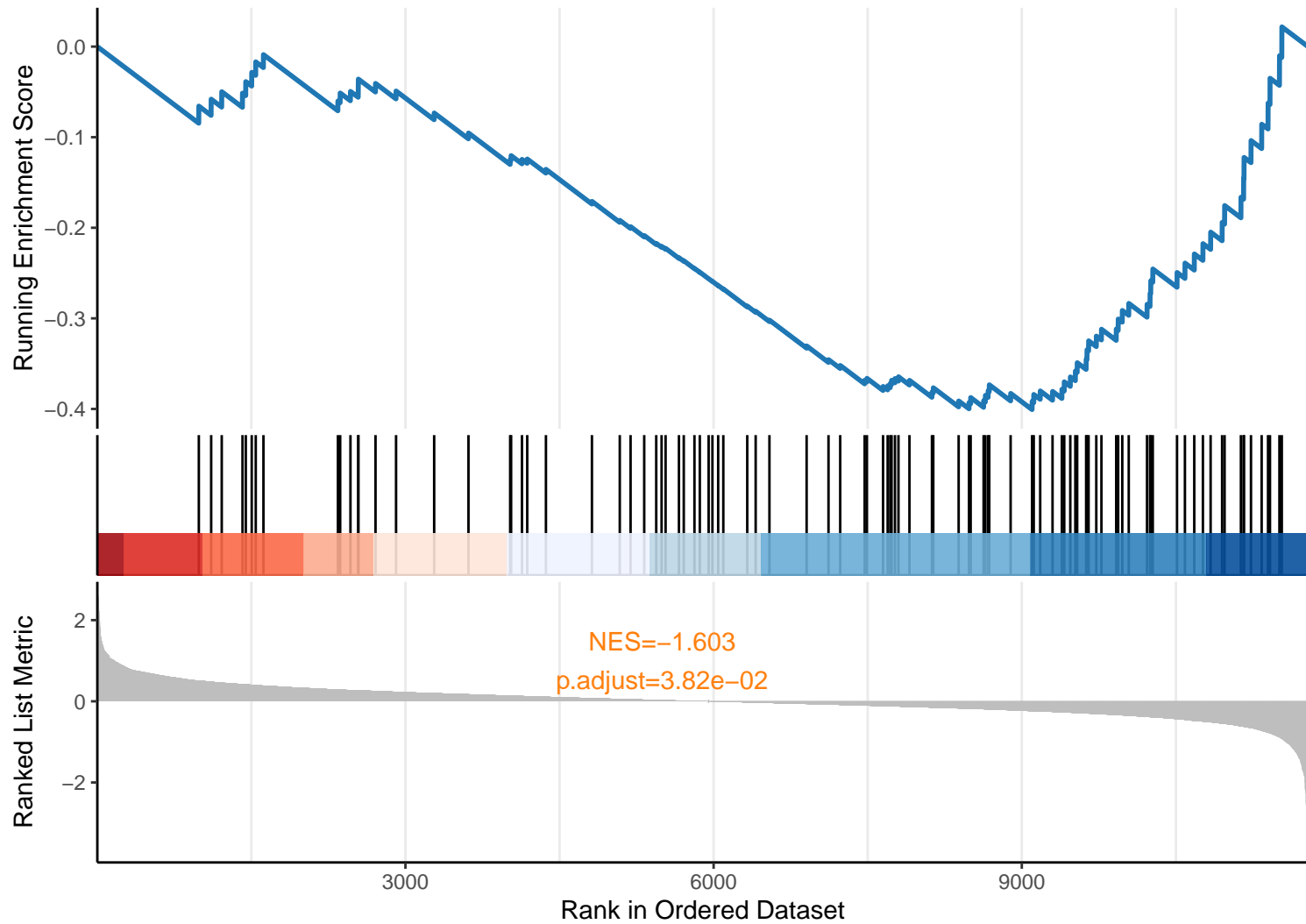

Supplement: Supplementary file 1 — Data S1. Supporting Information. [file AME2-7-781-s001.zip › Supplementary Material 4/KEGG_SPLICEOSOME_gseaplot.pdf]

# KEGG\_VEGF\_SIGNALING\_PATHWAY

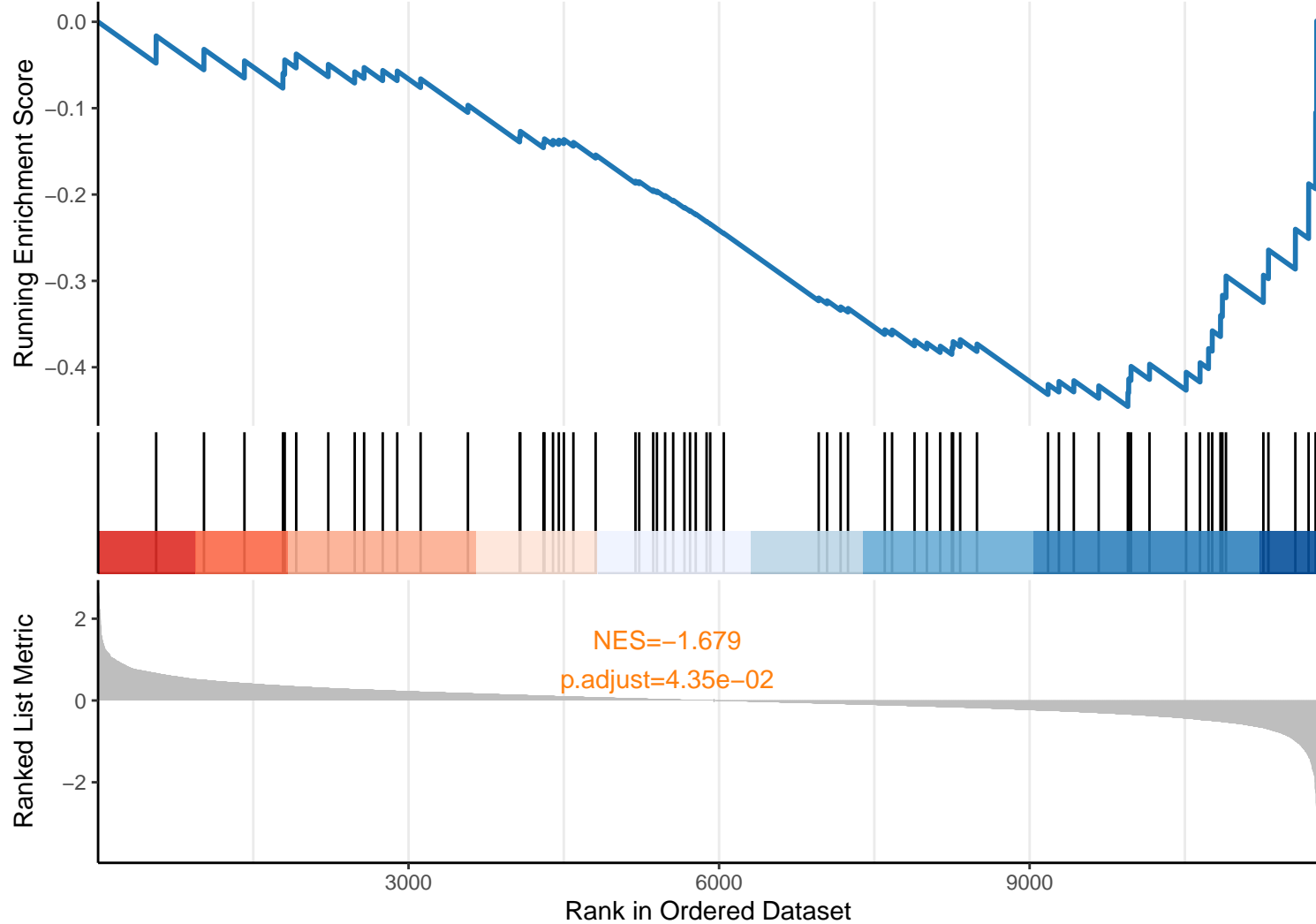

Supplement: Supplementary file 1 — Data S1. Supporting Information. [file AME2-7-781-s001.zip › Supplementary Material 4/KEGG_VEGF_SIGNALING_PATHWAY_gseaplot.pdf]

# KEGG\_VIRAL\_MYOCARDITIS

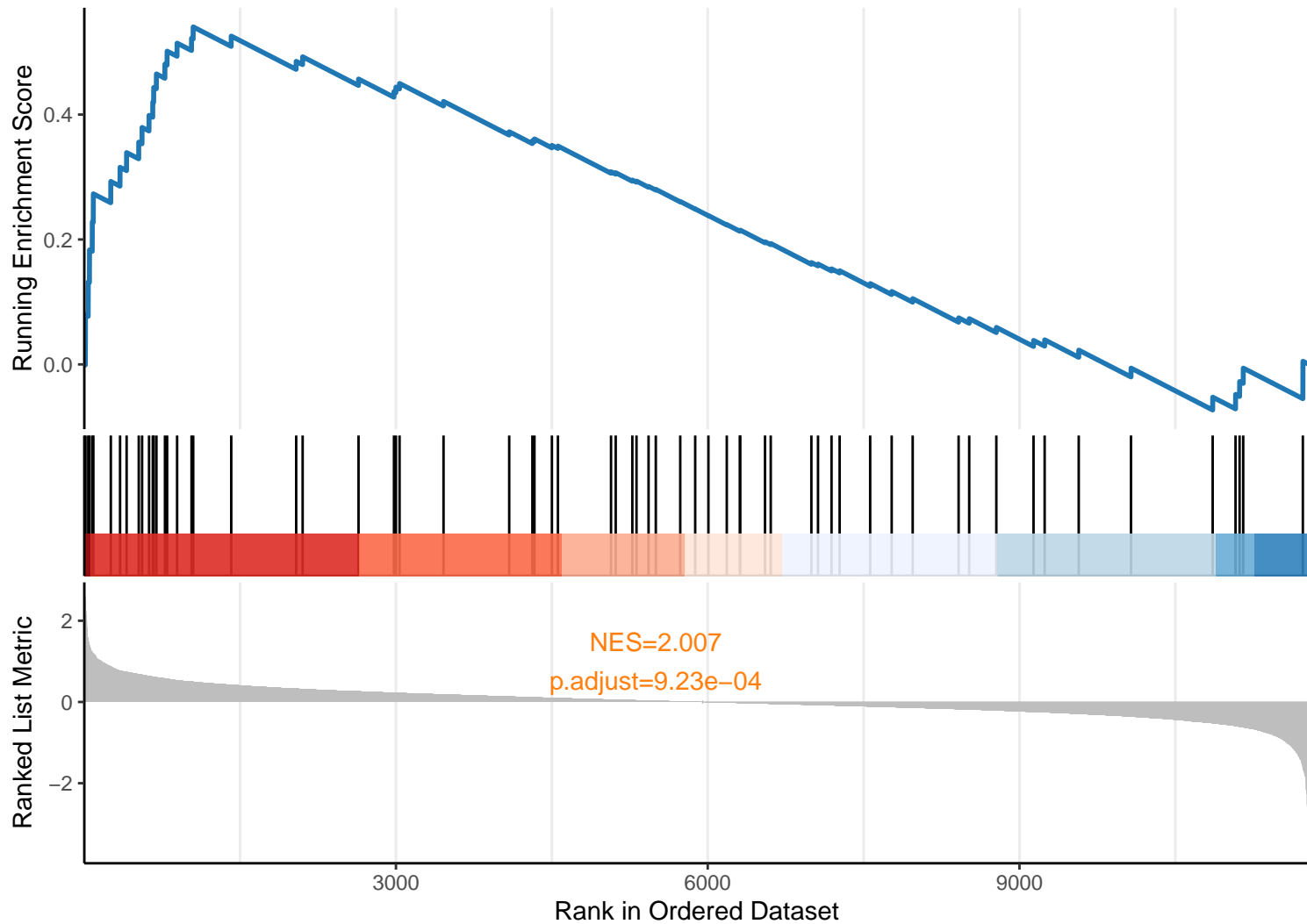

Supplement: Supplementary file 1 — Data S1. Supporting Information. [file AME2-7-781-s001.zip › Supplementary Material 4/KEGG_VIRAL_MYOCARDITIS_gseaplot.pdf]
